# Medical Appointments

**Published:** 1842-09

**Authors:** 


MEDICAL APPOINTMENTS.

Dr. John Moorehead has been selected to fill the chair of Theory and Practice of Medicine in the Medical College of Ohio, vacated by the resignation of Dr. J. P. Kirtland. Dr. Moorehead was for several years Professor of Obstetrics in the same institution; he also held at one time the situation to which he is now again called, during which period, if we are correctly informed, he lectured for the space of *six weeks* on *Coprostasis*. Should the worthy professor do the same thing the coming winter, we hope our friend, the doughty knight of the Western Lancet, will flesh his instrument in him.

Dr. N. Worcester has been appointed Professor of *Physical Diagnosis* and *Pathological Anatomy*, a new chair created expressly for him in the same school. The faculty of this college is now complete. C.

